# Global Elderly Migrations and Their Impact on Health Care Systems

**DOI:** 10.3389/fpubh.2020.00386

**Published:** 2020-08-25

**Authors:** Tomasz Holecki, Anna Rogalska, Karolina Sobczyk, Joanna Woźniak-Holecka, Piotr Romaniuk

**Affiliations:** ^1^Department of Health Economics and Health Management, School of Health Sciences in Bytom, Medical University of Silesia in Katowice, Bytom, Poland; ^2^Department of Health Promotion, School of Health Sciences in Bytom, Medical University of Silesia in Katowice, Bytom, Poland; ^3^Department of Health Policy, School of Health Sciences in Bytom, Medical University of Silesia in Katowice, Bytom, Poland

**Keywords:** globalization, aging, migration, health care systems, public health

## Abstract

Demographic transitions that occur in decreased dynamics of natality and rising number of elderly in population structures constitute a challenge for all national economies. Another global phenomena are large-scale migration processes driven by intensification of globalization process, development of technologies, and telecommunications. Although both these phenomena were vastly addressed in many ways in scientific literature, a notifiable fact is that there are only few researches that would investigate them in connection and consider migration of older people and its consequences, especially for health systems. Despite the fact that generally the likelihood of migrations reduces along with age, in some countries a higher share of migrants older than 65 years in reference to the entire group of migrants are being observed. It is the more essential that groups of seniors represent an increasing percentage of people. There are also differences in between standard reasons for migrations in young people and the factors affecting migrations in elderly ones. Many variables can influence migration decisions among older people, and they can be affected by seniors' health conditions, levels of health care within the target countries they migrate to, and the living standards. Such factors as population aging, reduced fertility, and international migration have affected the changes in demographic profiles of many countries. The consequence of migration decisions in the group of seniors is, among others, the impact on health care systems of single nation states, which are more and more important elements of economic, social, and financial systems.

## Introduction

Economic globalization use to be dated by some researchers back to the bronze age, where the first trade relationships took origin (1). However, 1970s and 1980s are considered to be extremely vital for this phenomenon in view of evident acceleration of multidirectional flows (2). The term “globalization” is used in reference to the intensification process of mutual relationships among societies in political, economic, cultural, and social dimensions. It occurs as an inherent part of the process initiated by expansion of trade, exploration, conquest, migrations, and colonization and bolstered by advance in technologies (3). Undoubtedly globalization as a process of integration practically affects every single aspect of life of individuals and societies (4).

One of the aspects of economies getting globalized is ease of traveling or changing a place of residence. In 2017, the number of people living in a country different from their homeland reached 258 million, which represented 3.4% of the global population (5). For comparison, in the year 2000, these figures came to 173 million, whereas in 2015, they amounted to as much as 244 million, which shows a clear 41% growth in 15 years. In terms of geographical distribution, ~31% of migrants of the global population reside in Asia, 30% in Europe, 26% in both Americas, 10% in Africa, and 3% in Australia and Oceania. Considering an economic factor only, migration can be viewed as investment in which an individual calculates a current discounted value of expected flow of earnings throughout his/her lifetime both in the case of changing one's place of residence and staying in current place of residence. In the next step, the individual makes a decision whether to migrate or not if returns lessened by migration costs are higher in the place of future residence (6). If the decision to leave a home country has been made, there comes a change that involves long-lasting effects not only for individuals and groups of people who are willing to migrate, but also for their countries of origin, destination, and even transit ones. Naturally, the biggest challenges need to be faced by the host countries, which need to face issues related to the management of the numbers and profiles of incoming migrants and modification of economic and social factors (6).

Migrants who make decisions to leave their home countries, no matter what the reasons are, always try to improve the quality of their lives. They may be attracted by better prospects and perspectives for development and higher living standards or social care level (7). One of the social groups whose members more and more often take decisions to change their countries of residence are seniors, who upon reaching the retirement age decide to emigrate because of socioeconomic living conditions–related reasons. All these phenomena bring new challenges also for health care sectors of single countries and their associations (7).

The aim of this article is to follow and monitor current trends that concern health care systems vs. more and more common decisions taken by seniors to change their countries of residence because of easier access to health care, a wider assortment of procedures offered to them to choose from, or a higher quality of services rendered. We also aim to provide perspective for future development of this trend, as well as its consequence for health systems globally.

## Data and Methods

As part of the study, we performed a secondary data analysis. We analyzed raw, existing, and previously archived data, which were collected by the Organization for Economic Cooperation and Development (OECD). We made a retrospective interpretation of the analyzed data, which made it possible to show the number of health care professionals acting on the market of health services, health care system index, and cost-of-living index in respective countries in the context of global elderly migration. We have fully evaluated the data set in terms of its completeness and quality, including the manner and period of collection. The OECD Health Statistics offers the most comprehensive source of comparable statistics on health and health systems across OECD countries and includes data found in the publication Health at a Glance. Over the years, the OECD Health Statistics database has been used in a number of analytical applications both within and outside the OECD. This database includes 37 OECD and 7 non-OECD countries. Our analysis covered 29 OECD countries because of the lack of data on the measurements of interest to us for the remaining eight countries.

The issue of elderly migrations and its determinants has been drawn based on the selection of studies identified in available scientific databases, which critical analysis, along with juxtaposition with abovementioned secondary data, enabled us to propose a set of conclusions. The limitation of the study is, however, the lack of extensive thematic studies on the migration of older people and their impact on the health care systems in country of residence. Therefore, some of the conclusions were drawn based a comparison between available databases in both the socio-functional and financial-organizational aspects.

The databases we searched to identify relevant literature were as follows: EMBASE, MEDLINE, Cochrane Library, and Google Scholar. Articles in journals were included if they reported the reasons or directions of older people migration flows, or the impact of older people's migration on health care systems. The number of publications on this topic was too small to justify a meta-analytical review.

### The Issue of Migrations in Older People

In the year 2017, the number of people older than 60 years was 962 million, and as estimated in 2050, it will go up to 2.1 billion, which will represent 20% of the population worldwide (7). In some countries, the share of people at 65 years or older has already exceeded 20% of their population. The examples of these are Bulgaria (20.4%), Finland (20.5%), Germany (21.1%), Italy (22%), and most significantly Japan (27.7% of the population in general) (8, 9). The process is connected with a long-term decline in fertility accompanied by extending life expectancy. This is a phenomenon that is expected to increase; hence, countries must be prepared for its inevitable consequences.

The present-day demographic situation related to the increasing number of older people has its important implications for economic systems. On the one hand, it raises concerns about how to meet new needs and how to finance them effectively. On the other hand, it brings a potential for development for many new branches of industries and service sectors by offering modern products and services dedicated just to this age group.

For many countries, one of challenges connected with the aging of societies in general is an increasing migration among older people. It is estimated that by 2050 almost one in five senior Americans (19%) will have had their place of birth outside the country (6). The global percentage of international migrants at 65 years or older was 30 million (11.7% of the total) in 2017 (10). The share of migration in seniors is higher in developed regions (13%) than in middle- and low-income countries (8%) (6). An estimated number of older migrants 65 years or older increased by more than 11 million in developed regions in the years 1990–2017, whereas in developing regions the number mentioned went up merely by less than a million. It results from the fact that migrants living in the southern hemisphere, as they get old, tend to go back to their countries of origin (7).

Migrations of elderly people are not structurally identical, making ground for distinguishing regional migration centers of different characters. The key region in North America to which elderly people emigrate is the United States (>6 million). By contrast, the countries in Western Europe that have the biggest stock of migrants 65 years or older are Germany, France, and Great Britain (6 million people in total) (8). However, in 2014, in Germany, among people who were older than 65 years, 7,500 more seniors emigrated from the country than emigrated to it, and the loss of population appeared both in German citizens (2,500 people) and foreigners living there (5,000 people) (11). In Great Britain, the percentage of people 65 years or older constitutes 19% of migrants (12).

In general, the existing data on older people migration directions, scale, and reasons are limited. In Poland, the number of foreigners with valid residence permits who reached the age of 65 or older came mainly from Germany (1,844) and then Italy (547), Sweden (283), and Great Britain (269). Among older British people, migration to rural and coastal areas in Great Britain has become a common phenomenon. In Germany, it was shown that the incentive for migration of older people, apart from family factors, was the low cost of living. Research in Malawi has shown a similar relationship, which clearly links individual health and the decision to migrate among older people. An additional factor attracting migrants is the insurance model of health care, in which costs of care on the side of the persons concerned are significantly reduced (8).

In this context, collecting and analyzing data on the demographic structure of migrants appear to be priority issue for health systems, which face a perspective to be highly affected by the migration of older people generating significant demand for specific health care services and new infrastructure of the host country. This seems fundamental for the preparation of action plans in the financial and management areas.

## Results

**Figure 1:**
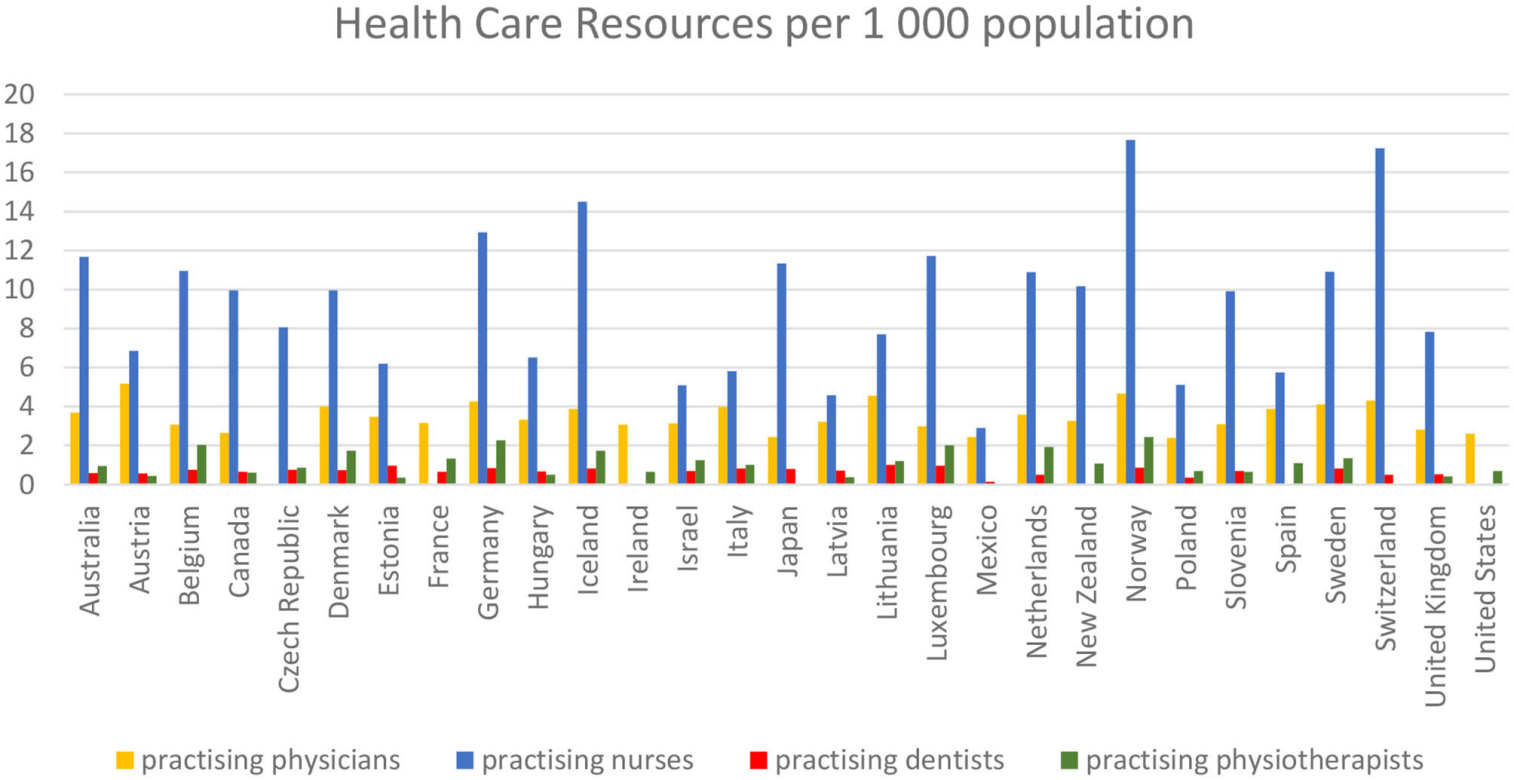
Number of practicing physicians, practicing dentists, practicing nurses, and physiotherapists per 1,000 inhabitants in respective countries in year 2017. Source: Own elaboration on the basis of (13).

As part of the study, we reviewed data on the number of practicing physicians, practicing dentists, practicing nurses, and physiotherapists per 1,000 inhabitants in respective countries in 2017 year. Figure 1 shows that the biggest number of physicians per 1,000 inhabitants occurs in Austria (5.18), Norway (4.66), and in Lithuania (4.56). The highest ratio of nurses occurred in Norway (17.67), Switzerland (17.23), and in Germany (12.93). The largest number of dentists was noted in Lithuania (1), in Luxembourg (0.97), and Estonia (0.96). The most of physiotherapists was employed in such countries as Norway (2.43), Germany (2.27), and Belgium (2.02).

**Figure 2:**
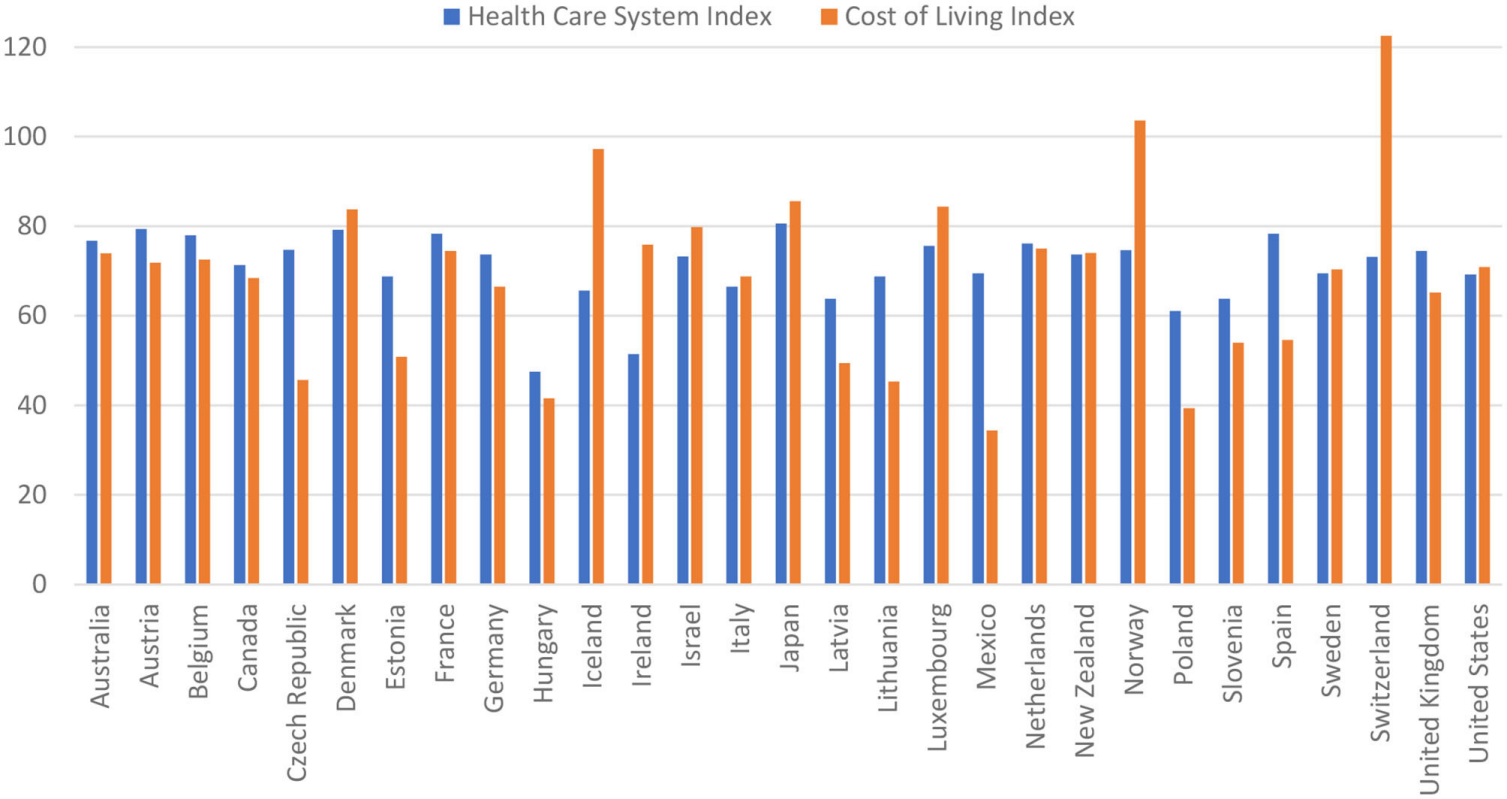
Comparison of health care system index with cost-of-living index in respective countries in year 2017. Source: Own estimation based on (14).

Second, we dealt with the comparison of health care system index with cost-of-living index in respective countries. Using this comparison, the lowest living cost index was noted in Mexico (34.39), Poland (39.34), in Hungary (41.53), Lithuania (45.33), and Latvia (49.39). Another crucial factor is the quality of services offered by the system and measured with the health care index, which occurred in the highest values in Japan (80.6), and among the European countries in Austria (79.33), Denmark (79.22), Spain (78.34), France (78.32), and also in Belgium (77.94). Details are presented in Figure 2.

## Discussion

Population of seniors is not a homogeneous group, differing in terms of physical fitness, somatic–mental health condition and socioeconomic situation (15). In results, migration of older people is usually accompanied by a strong, but not always positive motivation. Older people definitely make decisions to emigrate less frequently compared to younger ones. One of the crucial reasons for this is their physical inability and mobility limitation. Of significance is also the fact of seniors' attachment to their home country or home soil. Many older people have invested huge amount of their time and money in their homes, and thereby they declare very strong bonds with their places of residence (16). There are also fears of starting a new life in another country, as well as a desire to secure the assets (17). In most cases, an identity of an individual is also connected to a certain geographical location (18), especially in the rural regions (18, 19), which is an obvious barrier to migration processes. The World Health Organization also promotes idea of getting old in a place of residence in order to avoid possible emotional disturbances connected with leaving homes and better control of institutional care costs. The fact that aging in a place of residence is economically, socially, and emotionally profitable prompts decision-makers and health service providers to prefer such a solution as well (20).

Older people not always, however, decide to stay in a place of permanent residence, and their most frequent causes of changing it remain different from those that induce young people to migrate. Litwak and Longino enumerated three migration groups among the elderly: those starting to retire, those who experience moderate forms of disability, and those who experience severe forms of chronic disability (21). Many other variables including, as already mentioned, expected facilities related to climatic conditions, recreation areas, natural resources (lakes, forests, and parks), and even antiquities that occur in a given area may also affect the migration decision (22, 23). Furthermore, expenses for health care matter when taking decisions by older people whether to migrate or not (24). The increase in number of the oldest seniors may then imply the increase in number of migrations “forced” by poor health conditions of potential migrants (25). In the study by Kaluza–Kopias on the representative sample of Poles aged 75 years or older, as many as 88% of them declared reluctance for relocations, and a desire among them to migrate in the future was mainly driven by health concerns, and to a lesser extent by such factors as attractiveness of the neighborhood to live in (16.8%) (25). A number of other studies in Europe also showed that international migrants more frequently than other people judged their health conditions as poor (26), although a country of origin is considered to be an important factor determining differences in health conditions among the population of migrants and the way they use health services (27).

Studies conducted in France showed that factors such as location, cultural, and environmental amenities, a high quality of rendered public and private public services, and low crime rates mattered in seniors when taking decisions whether to migrate or not within a country and beyond it (28). The decisions connected with migration of seniors may be therefore derived from a combination of various factors, but one of the key motivators appears to be a comfortable access to medical care, where one of the determinants of which is the number of health care professionals acting on the market of health services, based on the data we used to conduct this analysis, as presented in the previous section.

Studies on the migrations of seniors show also that they are particularly willing to settle in larger cities, where the living conditions are more favorable, and the access to technical infrastructure and medical services is easier (29). Additionally, there is also a trend to settle in nursing homes after migrating to a new area. McAuley, Pechioni, and Grant proposed the concept that the entire process of seniors' migration is grounded in the decision-making process related to long-term care (26). According to this hypothesis, seniors who have not found a suitable institution near their place of residence move or are deployed by families to completely new locations. Because of the differences in cost of living, the trend of sending older family members outside the home country is becoming more pronounced. This is favored by extensive communication networks, both traditional, such as highways, rail, or air connections, and the modern technologies that facilitate contact via the Internet or mobile devices.

Our hypothesis that access to adequate healthcare is one of the most important drivers for the elderly migrations seems to be confirmed in light of the existing studies on this topic, especially when combined with selected data from the OECD countries regarding availability and quality of healthcare. However, this is still not enough to make adequate financial and organizational decisions regarding health care systems. To provide sufficient foundations for this kind of intervention, further in-depth research of this submarket and its features is needed, and we believe the analysis we presented in this article will be a starting point for these further studies.

## Concluding Remarks

If to analyze migration flows based on the Healthcare System Index, it appears that patients and their families are looking for countries with higher or similar level of development of health services. However, in the population of seniors not only the access to health care does matter, but also a combination of a few other factors, including their readiness to meet families who have emigrated before. The real living expenses measured with the purchasing power of currency is also of importance.

Intensification and migration trends of numerous or homogeneous population groups are very important from the standpoint of the economic and health security of the countries bringing in emigrants. It is obvious that a growing migration trend among the elderly has its consequences, as it produces a financial burden for health care systems in destination countries, as well as it involves epidemiological risk. Putting the hypothesis that seniors make a decision about migration based on a combination of factors such as the quality and availability of medical services and the simultaneous low or average cost of living, the main directions of migration among older people should be countries such as Denmark, Austria, France, and Germany. Hence, it seems to be necessary to introduce with respect of civil rights such supranational systemic solutions not to burden single countries or regions with migration problems.

Presented issues should be preceded by more intensive analyses in order to provide relevant scientific evidence to take action to optimize solutions in health systems. Nevertheless, the basic conclusions can be considered as follows:

The migration process among seniors relies on a number of complex factors underlying their final, often irrevocable decision. The most important of these factors are lower maintenance costs, access to properly adapted transport and infrastructure, and access to high-quality health care in connection with the relatively low price of products and services offered on this market;

Migration of older people is particularly important for the economies of the host countries due to the rising population of people in retirement age, with simultaneous acceptance of inevitability to secure access to the health care system to a significant number of people with a very high individual rate of service consumption;

Economic forecasts regarding further intensive development of globalization process should be considered as referring also to the intensified senior migration. It is necessary to implement measures boosting its positive impact on the national economies, while seeking ways to minimize the occurring negative effects, especially by making preparations in terms of health care system infrastructure, or seeking for new cost-effective ways of delivering health services under conditions of increased demographic burden;

Effective senior policy should take into account the implementation of innovative solutions at the local, governmental, and supranational level. This is all the more important that this aspect is increasingly affecting the condition of health care systems and may have impact on the stability of extensive economic areas, which in turn may be crucial for ensuring financial stability of health systems loaded with increased burden;

Migration of elderly outside the country of residence should also be seen as a risk of recurrence of old or spread of new, unknown epidemics in a given country. These risks, as well as seniors' multimorbidity and their greater susceptibility to infection, should also be taken into consideration when adjusting health systems to the new reality determined by migration processes.

## Data Availability Statement

All datasets generated for this study are included in the article/supplementary material.

## Author Contributions

TH conceived the study and prepared final version of the paper. AR contributed to introduction and discussion sections in the draft version of the paper. KS collected the data and performed all analyses in the draft version of the paper. JW-H collected the data and contributed to the results section in the draft version of the paper. PR contributed to the results interpretation and conclusions in the final version of the paper. All authors contributed to the article and approved the submitted version.

## Conflict of Interest

The authors declare that the research was conducted in the absence of any commercial or financial relationships that could be construed as a potential conflict of interest.
